# HOTAIR contributes to the carcinogenesis of gastric cancer via modulating cellular and exosomal miRNAs level

**DOI:** 10.1038/s41419-020-02946-4

**Published:** 2020-09-19

**Authors:** Jie Zhang, Wei-qing Qiu, Hongyi Zhu, Hua Liu, Jian-hua Sun, Yuanwen Chen, Huojian Shen, Chang-lin Qian, Zhi-yong Shen

**Affiliations:** Department of General Surgery, South Campus, Ren Ji Hospital, School of Medicine, Shanghai Jiao Tong University, 201112 Shanghai, China

**Keywords:** Gastric cancer, Preclinical research

## Abstract

Gastric cancer (GC) is one of the most leading malignancies. Long noncoding RNA is related to GC. In this study, 11 miRNAs in the exosomes and six lncRNAs in the tissues was examined by qRT-PCR. Correlation analysis was used to analyze the relationship between miRNAs in exosome and lncRNAs in the tissues. Four miRNAs level in GC tissues were examined by qRT-PCR. MTT was used to determine cell viability. Flow cytometry was used to quantify the apoptotic cells. Transwell assay was used to examine the migration and invasion capacity. Dual-luciferase assay was used to examine the interaction between HOTAIR and miR-30a or -b. Capillary formation was used to determine the capillary formation capacity. Weak negative correlations were found between HOTAIR and miR-30a or -b in GC tissue samples. Interestingly, strong negative correlations were identified between the HOTAIR level in GC tissue samples and the miR-30a or -b levels in plasma exosomes. HOTAIR knockdown GC cells exhibited decreased migration, invasion, proliferation, and upregulated apoptosis, which released more miR-30a and -b into the exosomes. KRAS was upregulated when co-cultured with exosomes from HOTAIR overexpressed cells, and promoted GC cells proliferation, migration, and invasion. Meanwhile, HUVEC cells expressed increased VEGF-A and formatted more capillaries. Subsequently, we identified a 10mer target site of miR-30a or -b in HOTAIR sequence, and the overexpression of HOTAIR induced the degradation of miR-30a or -b, indicating a ceRNA role of HOTAIR. We report the negative correlation between the plasma miRNAs level and GC tissue HOTAIR expression for the first time and unveiled the ceRNA role of HOTAIR in GC. HOTAIR functions as an onco-lncRNA regulating the level of miR-30a and -b in both GC cells and exosomes. These findings may give insight into understanding the mechanism of GC pathogenesis and provide new biomarkers for clinical diagnosis.

## Introduction

Gastric cancer (GC), also known as stomach cancer, is one of the most common cancer in the world^1–3^. Due to the advancement of medical technology, the mortality of gastric cancer have gradually decreased, but there are still nearly 700,000 confirmed mortalities annually worldwide^4^. Furthermore, a large number of patients are diagnosed with advanced gastric cancer and have a poor prognosis. Thus, more sensitive markers for GC early stage diagnosis, tumor grading, prognostic evaluation after treatment is urgently needed.

MicroRNAs (miRNAs) are a group of gene expression negative regulators, which repress gene expression at post-transcriptional level by binding to the 3′-untranslated region (UTR) region of target mRNA directly. MiRNAs are involved in gene function modulation during many different biological processes, such as proliferation, apoptosis, and differentiation. It has been verified that miRNA expression profile is altered during the initiation, progression, and metastasis of human cancers and it is believed that miRNAs may function both as tumor suppressors and oncogenes in cancer development^5,6^. miRNAs alteration has also been found in the serum samples of GC patients, which has the potential to be used as biomarker for GC diagnosis and classification.

Exosomes are a kind of extracellular vesicle (EV), the diameter of which ranging from 40–100 nm, secreted by cells, and transfer signal molecule to targeted cells. Tumor-derived exosomes containing key regulators modulating the process of tumor migration, metastases, angiogenesis, and so on. Meanwhile, a group of miRNAs was found packaged by exosomes and exosomal miRNAs’ level in serum has been confirmed relates to the development and prognosis of cancers^7,8^.

Recent genetic study indicates that more than 70% of the human genome is transcribed to RNA, but only about 1% is protein coding, suggesting that a large group of RNAs function as gene expression regulators regulating a relatively small amount of effectors^9–11^. Long noncoding RNA (lncRNAs) is a group of more than 200 nt non-protein coding RNA molecules, that have been identified as key regulators of diverse cellular processes including development, differentiation, and cell fate as well as disease pathogenesis^12,13^. lncRNAs can serve as enhancers or repressors directly modulating target genes expression; and they can also function as molecular decoys, scaffold and signal mediators^14^.

HOX antisense intergenic RNA(HOTAIR) is 2158 nucleotides long non-protein coding RNA, which is transcribed from the antisense strand of the HOXC gene cluster^15^. HOTAIR has been found as a oncogene overexpressed in both solid tumors and hematologic malignancies. In gastric cancer, HOTAIR expression was higher in tumor when compared with the adjacent noncancerous tissues, and was significantly correlated with lymph node metastasis, TNM stage, and invasion^16,17^. High HOTAIR expression was also used as a predictor of poor overall survival in patients with GC^18^. HOTAIR was shown to directly bind to and inhibit miR-126, thereby promoting the expression of vascular endothelial growth factor A (VEGF-A) and PI3-K regulatory subunit beta^19^. HOTAIR binds to the multiprotein chromatin modifying complex PRC2, which is responsible for H3K27me3, an epigenetic hallmark of repressed chromatin, thereby repressing further the expression of miR-34a in GC^16^.

It is reported in human cells and fruit fly that, the extensive base pairing between miRNAs and cognate target mRNAs promotes miRNA decay^20^. However, in vitro and in vivo assays with Caenorhabditis elegans showed that targets can also protect miRNAs from active degradation^21,22^. HOTAIR has been reported relates to abnormal miRNAs level in tumors, but whether the interaction between miRNA and HOTAIR induces instability of miRNAs was still unknown.

In this study, the level of 11 candidate miRNAs in the plasma exosomes and the expression of four lnRNAs in the GC tissue samples were detected in 87 GC patients. Negative correlation was found between the expression of 3 miRNAs and HOTAIR. We also identified HOTAIR functions as a ceRNA modulate the tumor microenvironment by control the level exosomal miRNAs.

## Materials and methods

### Patients and samples

A total of 87 specimens of primary gastric adenocarcinoma, corresponding adjacent nontumorous gastric tissue and plasma samples were obtained between 2012 and 2014 at Renji Hospital, Shanghai Jiao Tong University School of Medicine. Tissue samples were immediately snap-frozen in liquid nitrogen and stored in a refrigerator at −80 °C. About 0.1 g tumor and adjacent control tissues were selected for RNA extraction followed by qRT-PCR. Control plasma samples were obtained from 87 age and sex matched healthy participants. Tumor staging was determined according to the tumor–node–metastasis classification system of the American Joint Committee on Cancer, 7th edition^23^. Follow-up was performed every 6 months by specially trained staff according to standard epidemiologic procedures. All samples were Random selected. Patients and controls were acquired with informed consent, under the protocol approved by Renji Hospital, Shanghai Jiao Tong University School of Medicine research ethics committee. The clinical characteristics were shown in Table 1 and Table S1.

**Table 1:** The association of exosome miR-30a and -b level with clinicopathological features.

	miR-30a low (Exo)	miR-30a high (Exo)	*P* value	miR-30b low (Exo)	miR-30b high (Exo)	*P* value
Age	63.5 ± 7.9	59.7 ± 10.5	0.11	62.9 ± 10.8	60.5 ± 8.9	0.082
Gender						
Male	21	20	0.48	19	22	0.34
Female	27	19		26	20	
Depth of invasion						
T1–T2	12	18	0.0037	8	22	0.0007
T3–T4	41	16		37	20	
TNM stage						
I–III	49	27	0.0036	41	35	0.28
IV	2	9		4	7	
Lymphatic invasion						
Negative	27	9	<0.001	20	16	0.34
Positive	11	40		23	28	

### Plasmid construction

For HOTAIR overexpression, full length of 2364 HOTAIR sequence was amplified by RT-PCR and cloned into pcDNA3.1 vector between Kpn I and EcoR I sites. For dual-luciferase assay, wild-type or mutant HOTAIR sequence was cloned into pmirGLO vector, following firefly luciferase coding region.

To construct KRAS reporter vector, a 521 nucleotides segment of human KRAS 3′UTR containing the miR-30a/b target site was cloned into pmirGLO vector between Xho I and Xba I sites.

To construct VEGF-A reporter vector, full length of 1.9 kb VEGF-A 3′UTR sequence was cloned into pmirGLO vector, between Xho I and Xba I sites.

### Exosome isolation

EVs were extracted from cell culture supernatant or plasma sample using Puro Exo exosome isolation kit (101Bio, USA) following the manufacture’s instruction. Briefly, plasma sample was centrifuge at 3000 × *g* for 10 min at 20 °C to remove cells and debris, followed by centrifugation at 10,000 × *g* for 20 min. The supernatant was filtrated with a 0.22 μm filter and then mixed with 0.2 volume exosome precipitation reagent. After 2 h incubation at 4 °C, the sample was centrifuged at 10,000 × *g* for 30 min at 20 °C. The pellet was resuspended by PBS for further study.

### RNA real-time RT-qPCR

Quantitive RT-PCR analysis was used to determine the relative expression level of candidate miRNAs. Total RNA was extracted from clinical samples and cells, using Trizol Reagent (Invitrogen, Carlsbad, CA, USA) according to the manufacturer’s instructions. The RNA samples were treated using TURBO DNA-free kit to remove the DNA contamination and then the DNA contamination was assessed by agarose gel. The level of candidate miRNAs was determined by qRT-PCR using miRNA-specific TaqMan MGB probes (Applied Biosystems, Foster City, CA, USA) after single-stranded cDNAs being synthesized. The level of U6 snRNA was used for normalization. Each sample in each group was measured three independent times with three repeats each time for determining the level of miRNAs. An Invitrogen reverse-transcription reagent kit was used according to the manufacturer’s instructions for reverse-transcription of 2 μg total RNA into first-strand cDNA. The qRT-PCR (ABI StepOnePlus Real-Time PCR system, Applied Biosystems, Foster City, CA, USA) included use of the SYBR^®^ Green real-time PCR kit (Invitrogen, Carlsbad, CA, USA). The level of GAPDH was used as loading control for lncRNAs. Each sample in each group was measured in triplicate and the experiment was repeated at least three times for the detection of miRNAs.

### Cell culture

Human gastric cancer cell lines (SGC-7901 and BGC-823) were purchased from the Institute of Biochemistry and Cell Biology of the Chinese Academy of Sciences (Shanghai, China) and maintained in humidified incubator at 37 °C in a CO_2_ incubator in Dulbecco’s modified Eagle’s media (DMEM) or RPMI 1640 medium supplemented with 10% fetal bovine serum (FBS) and 1% penicillin–streptomycin.

### In vitro invasion assay

Indicated cells were resuspended at 5 × 104 cells per milliliter in serum-free DMEM and then seeded into to Matrigel-coated inserts (Becton Dickinson) and placed in 24-well trans plates with DMEM and 10% FBS (chemoattractant) to induce the cells invade through the pores to the trans side. After 12 h, the cells and Matrigel in the upper inserts were discarded, and the cells in the bottom trans chambers were fixed with 3% paraformaldehyde and stained with cryVRstal violet. The number of cells included in five randomly different fields were counted in. The experiments were performed in triplicate wells and each experiment was performed in triplicate.

### In vitro migration assay

Indicated cells were maintained in no-serum medium for 12 h and then treated with mitomycin-C. Cells were trypsinized and introduced into the upper chamber (1 × 10^5^/well) of the Transwell (8 µm pore size; BD Bioscience). The chemoattractant in the lower chamber was medium supplemented with 10% FBS. After 12 h, the cells that migrated to the lower chambers were fixed with 3% paraformaldehyde, stained with crystal violet. Crystal violet-stained cells were counted in five randomly different fields with an inverted microscope. The experiments were repeated for independent three times and each experiment was performed in triplicate.

### Dual-luciferase assay

For luciferase reporter assays, HEK293T cells were seeded in 48-well plates. miRNA mimics and luciferase reporter vectors were co-transfected into cells using lipofectamine 2000 (Invitrogen, Carlsbad, CA USA). Two days after transfection, cells were harvested and assayed with the Dual-Luciferase Assay kit (Promega, Madison, WI USA). Each treatment was performed in triplicate in three independent experiments. The results were expressed as the ratio of Firefly luciferase and Renilla luciferase.

### Cell proliferation assay

BGC-823 and SGC-7901 cells were seeded in 96-well plates at low density (5 × 10^3^) in DMEM culture, and allowed to attach overnight. The cells were then transfected with HOTAIR siRNA, with sequence scrambled short RNA as control. Twenty microliters MTT (5 mg/ml) (Sigma, St. Louis, MO, USA) were added into each well 48 h after transfection, and the cells were incubated for further 4 h. The absorbance was recorded at A570nm with a 96-well plate reader after the DMSO addition.

### Cell apoptosis assay

The apoptotic cells were quantified using flow cytometry after annexin V- fluorescein isothiocyanate/ prepidium iodide (PI) double staining (BD Bioscience).

### Capillary formation assay

0.1 ml growth factor-reduced Matrigel (BD Biosciences) was seeded into 96-well plate and solidified at 37 °C for 1 h. Indicated cells were then seeded on top of the Matrigel at 40,000 cells/well. Images of each well were taken at ×10 magnification using a invert microscope after 8 h of incubation at 37 °C. Each condition was assessed in triplicate. Tube formation was quantified using Imag J, which measured the total tube length, and the total number of tubes, loops, and branching points.

### Generation mouse xenograft model

All experiments involving animals were approved by Renji Hospital, Shanghai Jiao Tong University School of Medicine Animal Care and Use Committee. Female severe combined immunodeficient (SCID) beige mice were purchased from Charles River Laboratories at age 8 weeks with a weight between 20 and 25 g and were Random selected. A total of 5 × 10^6^ BGC-823 cells in 100 μL PBS together with an equal volume of Matrigel basement membrane matrix were injected subcutaneously into the shoulder to establish a human GC cell xenograft model. Exosomes were injected directly into the tumor at days 14, 21, 24, and 28 after tumor cell injection.

### Immunohistochemistry

Formalin-fixed, paraffin-embedded sections of tumor specimens were deparaffinized and then incubated with mouse anti-Ki-67 primary antibody at 4 °C overnight. After incubation with HRP conjugated goat anti-mouse sencondary antibody, the detection of signal was achieved using DAB Substrate kit following the manufacture’s instruction. Images were obtained using a microscopy (Leica). The slides were examined by three pathologists with the blind disciplines.

### Statistical analysis

Two-tailed Student’s *t*-test was used to calculate statistical significance between two comparator groups. The variance similarity was examined by F test. If the variance between groups is unsimilar, Welch’s test was used for correction. The differences of miRNA and lncRNA expressions between paired tissue sample was evaluated with Wilcox-matched pairs signed ranks test. The correlation analysis was analyzed by χ2-analysis. The differences between more than two groups were analyzed by One-way ANOVA. ALL the data were analyzed by using SPSS Statistical Package version 19. The survival times of different groups of patients were analyzed using the Kaplan–Meier method. A *P* value <0.05 was considered as statistically significant.

## Results

### HOTAIR is negatively correlated with miR-30a/b and miR-16 in the plasma and GC tissue samples

**Fig. 1:**
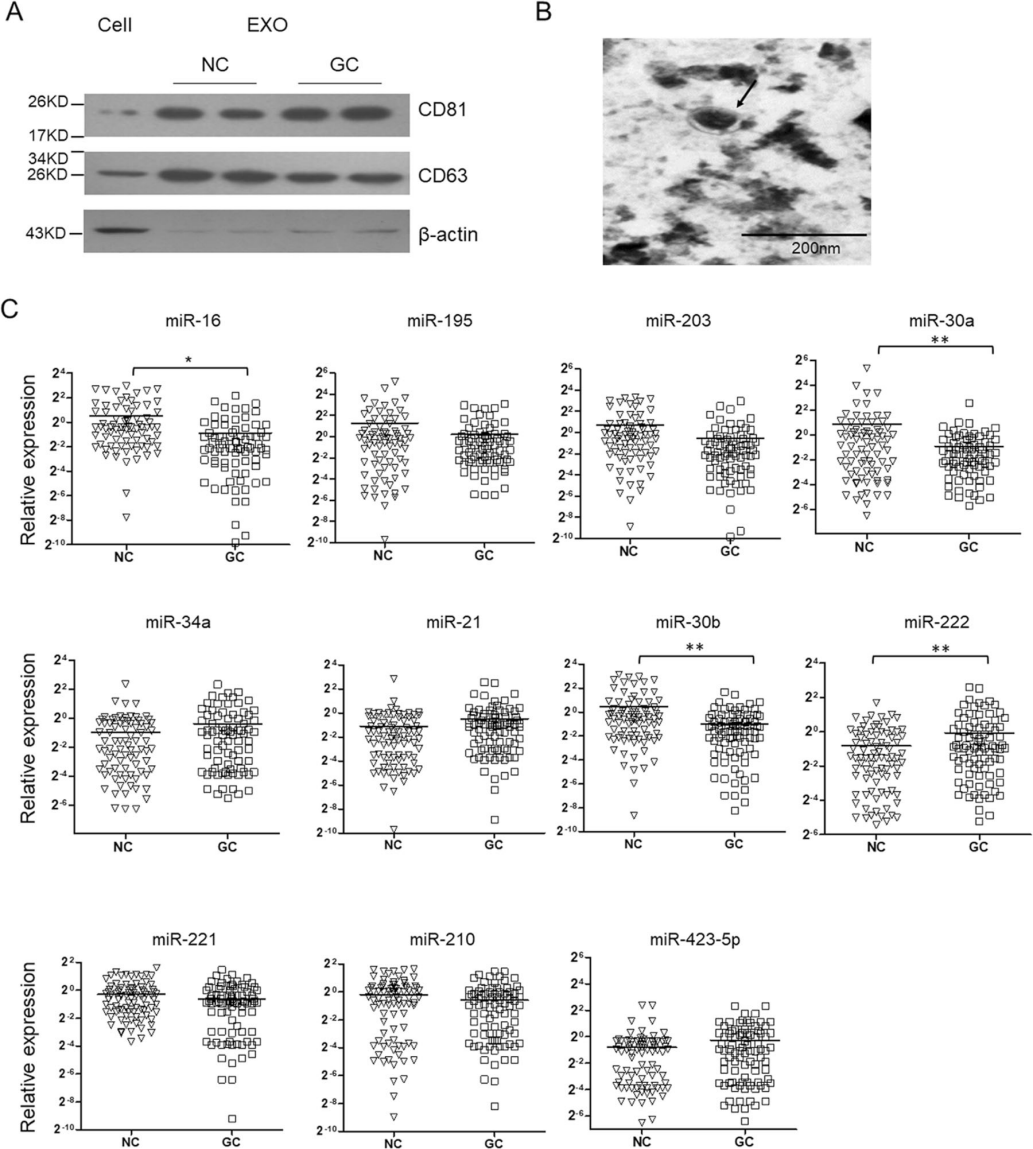
Plasma exosomal miRNAs level in GC patients. Exosomes were isolated from plasma samples of 87 GC patients by stepwise centrifugation. The isolation of exosomes was confirmed by detecting CD63 and CD81 by immunoblotting (**a**). **b** Electronic microscopic image of exosomes. **c** Total RNAs were extracted by Trizol reagents and miRNAs level were determined by qRT-PCR. Results were analyzed by student’s *t*-test and *P* < 0.05 was considered statistically significant.

**Fig. 2:**
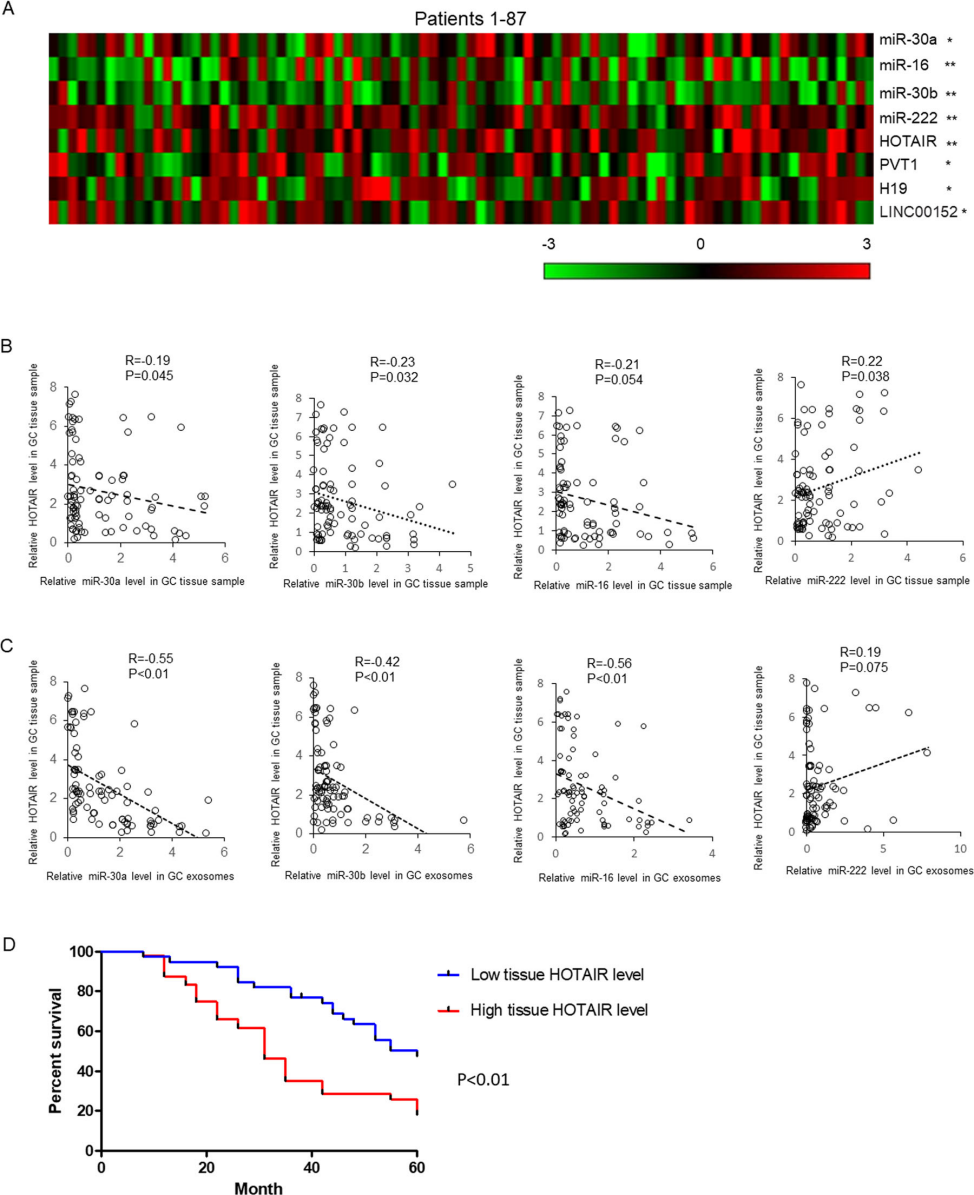
The level of miRNAs in GC tissue samples and the correlation analysis. **a** The expression of four miRNAs and four lncRNAs were detected in GC tissue samples and adjacent normal controls by qRT-PCR. The results were representing as heat map. Results were analyzed by student’s *t*-test and *P* < 0.05 was considered statistically significant. *P < 0.05, **P < 0.01. The correlations between tissue HOTAIR level and tissue (**b**) or plasma exosomal (**c**) miRNAs’ expressions were analyzed by χ2-analysis. **d** The overall survival curves are presented according to the expression level of HOTAIR in GC patients.

Extracellular miRNAs are mostly protected by extracellular vesicles especially exosomes and have been found in plasma, serum, urine, saliva, and milk. To understand the expression pattern of miRNAs in the exosomes from GC patients’ plasma samples, we first extracted total RNA from plasma exosome of 87 GC patients and healthy controls. The exosomes isolation was confirmed by detecting the protein markers(CD81 and CD63) (Fig. 1a), and morphology analysis using electron microscopy(Fig. 1b). We detected the level of 11 candidate miRNAs, the level of which changed in the GC patients especially in the blood samples^24,25^. As shown in Fig. 1, the level of exosomal miR-16, miR-30a and miR-30b was significantly downregulated in GC patients and the level of miR-222 was upregulated. Subsequently, the expression of miR-16, -30a, -30b, -222 and four lncRNAs in the GC tissues and adjacent noncancerous tissues was determined by qRT-PCR. As shown in Fig. 2a, the expression of miR-16, miR-30a, and -30b was significantly downregulated. Meanwhile, the level of HOTAIR, H19, PVT1, and LINC00152 was upregulated.

To understand whether there are some relationships between lncRNAs level and miRNAs level in exosomes or tissues, correlation analysis was employed. As shown in Fig. 2b, weak negative correlations existed between HOTAIR levels and miR-16, -30a, or -30b level (R = −0.21, *P* = 0.054; R = −0.19, *P* = 0.045; R = −0.23, *P* = 0.032). Meanwhile, HOTAIR level positively correlated with miR-222 level in GC tissues (R = 0.22, *P* = 0.038). Interestingly, strong negative correlations existed between HOTAIR level and exosomal miR-16, -30a or -30b level (R = -0.56, *P* < 0.01; R = −0.55, *P* < 0.01; R = −0.42, *P* < 0.01). After analyzing the survival rate of GC patients with high (49 patients) or low (38 patients) HOTAIR level in the tissue samples within 60 months, we found a significant low survival rate in the GC patients with higher HOTAIR level (Fig. 2d).

### HOTAIR binds with miR-16, miR-30a, and miR-30b, and induces their degradation

**Fig. 3:**
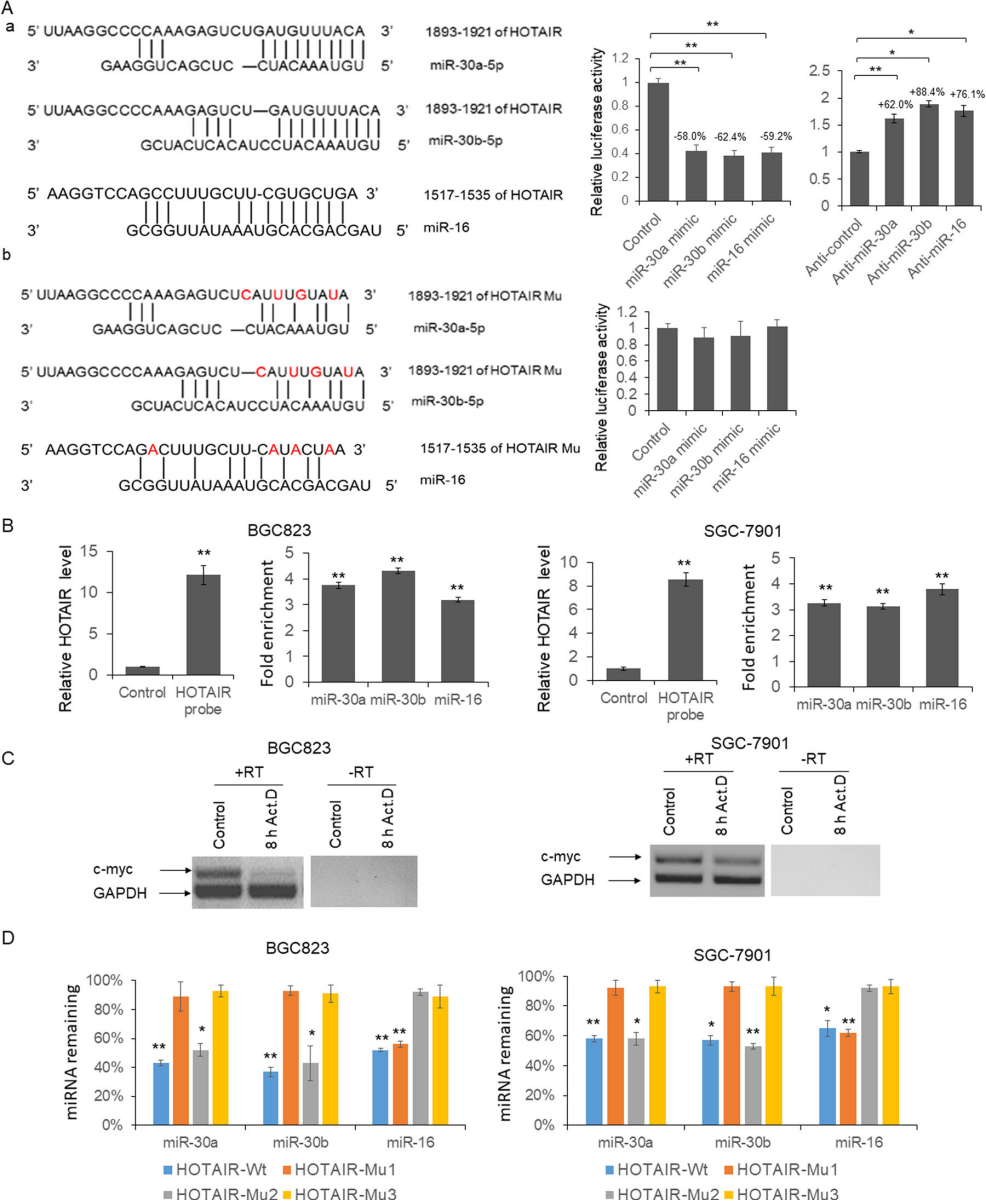
HOTAIR modulates the degradation of miR-30a, -b, and miR-16 through direct interaction. **a** a, schematic diagram of the predicted interaction between wild-type HOTAIR and miRNAs, and the dual-luciferase assay was used to confirm the interaction; b, schematic diagram of the predicted interaction between mutated HOTAIR and miRNAs. Results were analyzed by One-way ANOVA and *P* < 0.05 was considered statistically significant. *P < 0.05, **P < 0.01. **b** RNA pulldown. BGC-823 or SGC-7901 cell lysates were incubated with biotin labeled HOTAIR probe or control DNA oligo for 2 h, and then incubated with streptavidin beads for 1 h. Total RNA were extracted after four times washes and the level of HOTAIR and miRNAs was examined by RT-PCR. Results were analyzed by student’s *t*-test and *P* < 0.05 was considered statistically significant. **P < 0.01. **c** BGC-823 and SGC-7901 cells were treated with actinomycin D for 8 h. Total RNA was extracted and the level of c-myc and GAPDH were examine by RT-PCR. **d** BGC-823 and SGC-7901 cells were transfected with wild-type or mutant HOTAIR expression vector for 48 h followed by 8 h treatment with actinomycin D. The level of miR-30a, -b, and miR-16 was examined by qRT-PCR. Results were analyzed by student’s *t*-test and *P* < 0.05 was considered statistically significant. *P < 0.05, **P < 0.01.

Recently, HOTAIR has been reported to modulate miRNAs expression through direct interaction or altered promoter region histone methylation^26–28^. To explore the mechanism of strong negative correlations between HOTAIR level in the GC tissues and miR-16, -30a, and -b level in the plasma exosomes, we first predicted the direct interaction between HOTAIR and miRNAs using online bioinformatics tool: DIANA TOOLS (http://diana.imis.athena-innovation.gr/DianaTools/index.php). As shown in Fig. 3a(a), there is a 10nt interaction between HOTAIR and miR-30a/b, and a 7 nt interaction between HOTAIR and miR-16. To confirm the predicted results, the reporter vector was constructed by cloning the full-length HOTAIR sequence into the pmirGLO plasmid following the firefly luciferase coding region. miRNAs mimics or antagonists were transfected into HEK293T cells with HOTAIR reporter vector. 48 h after transfection, cells were lysed and the luciferase activities was detected. As shown in Fig. 3(a), the relative firefly luciferase activities were reduced to 58.0, 62.4, and 59.2% by miR-30a, -30b, and -16. When four nucleotides were mutated, the luciferase activities were not repressed by miR-30a, -30b, and -16 mimics (Fig. 3a(b)).

To further examine the negative correlation between HOTAIR and miRNAs, we knocked down and overexpressed HOTAIR in GC cell lines by HOTAIR siRNAs or expression vector. Meanwhile, HOTAIR pulldown assay was used to confirm the interaction between HOTAIR and miRNAs(Fig. 3b). We found HOTAIR probe successfully recruited HOTAIR in GC cells, while miR-30a, -b, and miR-16 were enriched more than three-fold by HOTAIR.

As shown in Fig. S1, the expression of miR-30a, -30b, and -16 was slightly upregulated by HOTAIR specific siRNAs in GC cells. Meanwhile, the exosomal miRNAs levels were sharply upregulated to more than three-folds, when HOTAIR was knocked down. Meanwhile, when HOTAIR was overexpressed in GC cells, the exosomal miR-16, -30a, and -30b levels were significantly reduced. These results indicated that exosomal miR-16, -30a, and -30b levels were precisely regulated by cellular HOTAIR level.

It is reported that the binding between target RNAs and miRNA regulated miRNA stability in human cells^20,29^. To understand whether HOTAIR regulates exosomal miRNA level through regulating miRNAs stability, three mutant HOTAIR expression vectors (HOTAIR-Mu1, miR-16 binding site mutation; HOTAIR-Mu2, miR-30a/b binding site mutation; HOTAIR-Mu3, miR-16, and miR-30a/b binding sites mutation) were constructed. BGC-823 and SGC-7901 cells were transfected with wild-type or mutant HOTAIR expression vector for 48 h and then treated with Actinomycin D for 8 h to inhibit RNA transcription. Total RNA from 8-h actinomycin D treated BGC-823 and SGC-7901 cells were RT-PCR amplified with primers to detect the unstable c-myc mRNA or stable GAPDH mRNA to confirm the transcriptional inhibition. As shown in Fig. 3b, a significant reduction in c-myc mRNA levels were found 8 h after transcription shutoff in both BGC-823 and SGC-7901 cells, while the GAPDH mRNA was still abundantly present. Meanwhile, the endogenous miR-30a/b level was dramatically reduced by HOTAIR-Wt and HOTAIR-Mu2, and the level of miR-16 was reduced by HOTAIR-Wt and HOTAIR-Mu1. These results indicated that the interaction between HOTAIR and miRNAs induced the instability of these miRNAs.

### HOTAIR repression inhibits GC cells proliferation, migration, invasion, and promotes apoptosis

**Fig. 4:**
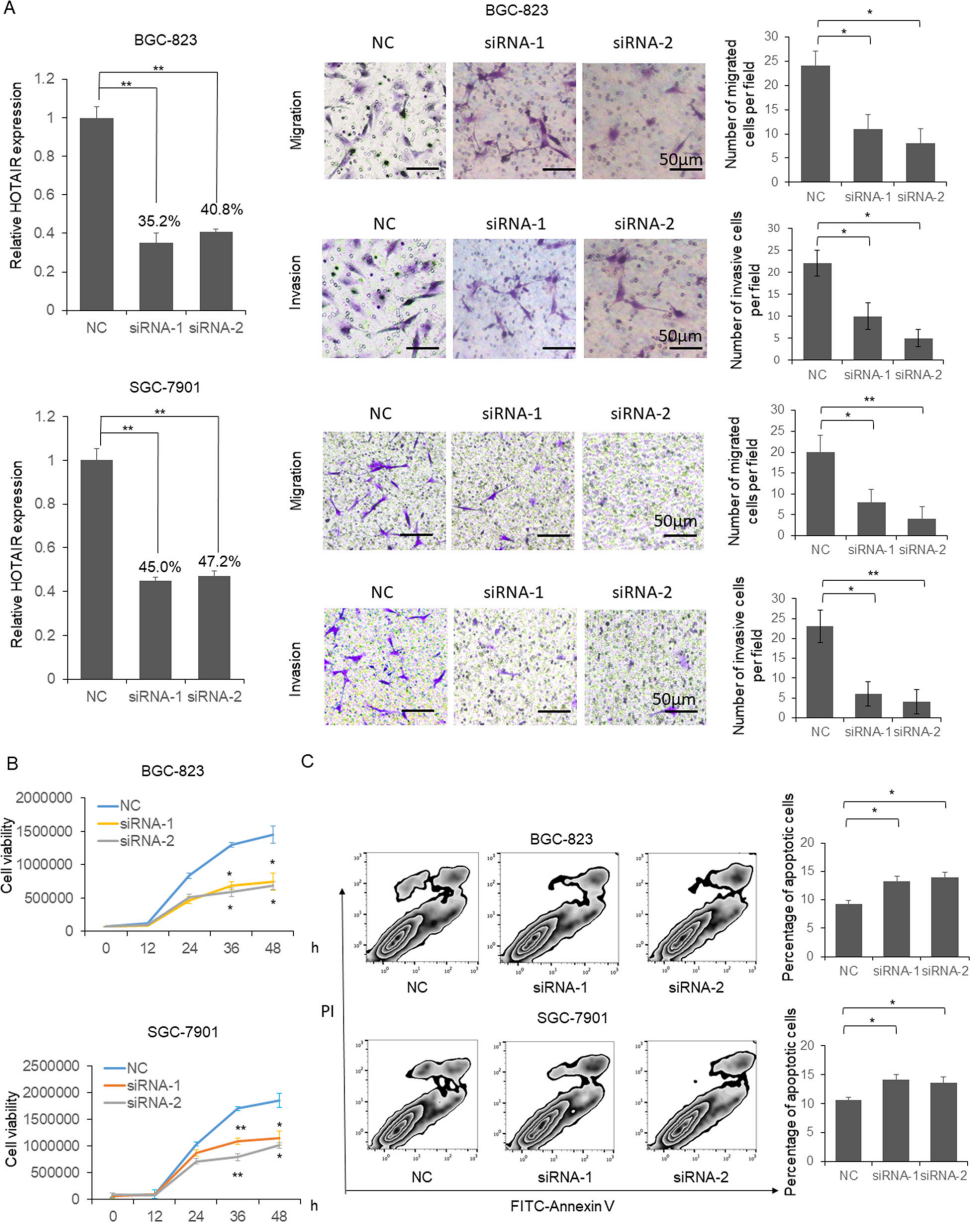
The biological function of HOTAIR in GC cells. **a** Two HOTAIR siRNAs were transfected into GC cells separately. 48 h after transfection, total RNA was extracted and the HOTAIR level was determined by qRT-PCR. In vitro cell migration and invasion assay were processed using traditional transwell plate. **b** MTT assay was used to determine the relative cell viability. **c** The numbers of apoptotic cells in HOTAIR knocked down GC cells were counted by flow cytometry after annexin V-FITC and PI staining. Results were analyzed by One-way ANOVA and *p* < 0.05 was considered as significant. *P < 0.05, **P < 0.01.

To explore the role of HOTAIR during the pathogenesis of gastric cancer, BGC-823 and SGC-7901 cells were transfected with two HOTAIR siRNA separately. Forty-eight hour post transfection, the HOTAIR level was determined by qRT-PCR. As shown in Fig. 4a left panel, the endogenous HOTAIR levels in BGC-823 and SGC-7901 cells were reduced to 35.2, 40.8, and 45.0%, 47.2% by HOTAIR siRNAs. Meanwhile, the cell migration and invasion capacity was also repressed by HOTAIR siRNAs when compared with sequence scrambled RNA control (Fig. 4a right panel). In the same time, cell viability and apoptosis were detected by MTT assay and flow cytometry. As shown in Fig. 4b, the cell viabilities were significantly reduced in BGC-823 and SGC-7901 cells by two HOTAIR siRNAs at 36 and 48 h post transfection. Meanwhile, apoptosis cell number was significantly upregulated in the siRNAs treated BGC-823 and SGC-7901 cells (Fig. 4c).

### HOTAIR promotes GC cells proliferation, migration, invasion, and inhibits apoptosis through protecting KRAS from the repression of miR-30a/b

To understand whether miR-30a/b regulated the HOTAIR function in GC cells, KRAS (confirmed miR-30a/b target gene) reporter vector was transfected into BGC-823 and SGC-7901 cells with or without HOTAIR expression vector or miR-30a and -b mimics. The cells were subjected to luciferase assay and immunoblotting to detect the KRAS expression. As shown in Fig. S2A, HOTAIR upregulated the luciferase activity and KRAS expression in both BGC-823 and SGC-7901 cells. Meanwhile, the mixture of miR-30a and -b totally repressed the function of HOTAIR on KRAS expression. Subsequently, cell viability, apoptosis, migration, and invasion assays were used to determine the biological function of HOTAIR and miR-30a/b in GC cells. The results indicated (Fig. S2B), inhibit apoptosis (Fig. S2C), promoted migration (Fig. S2D), and invasion (Fig. S2E) in both BGC-823 and SGC-7901 cells. Meanwhile, the mixture of miR-30a and -b totally repressed the function of HOTAIR.

### HOTAIR controls functional miR-30a/b level in exosomes

**Fig. 5:**
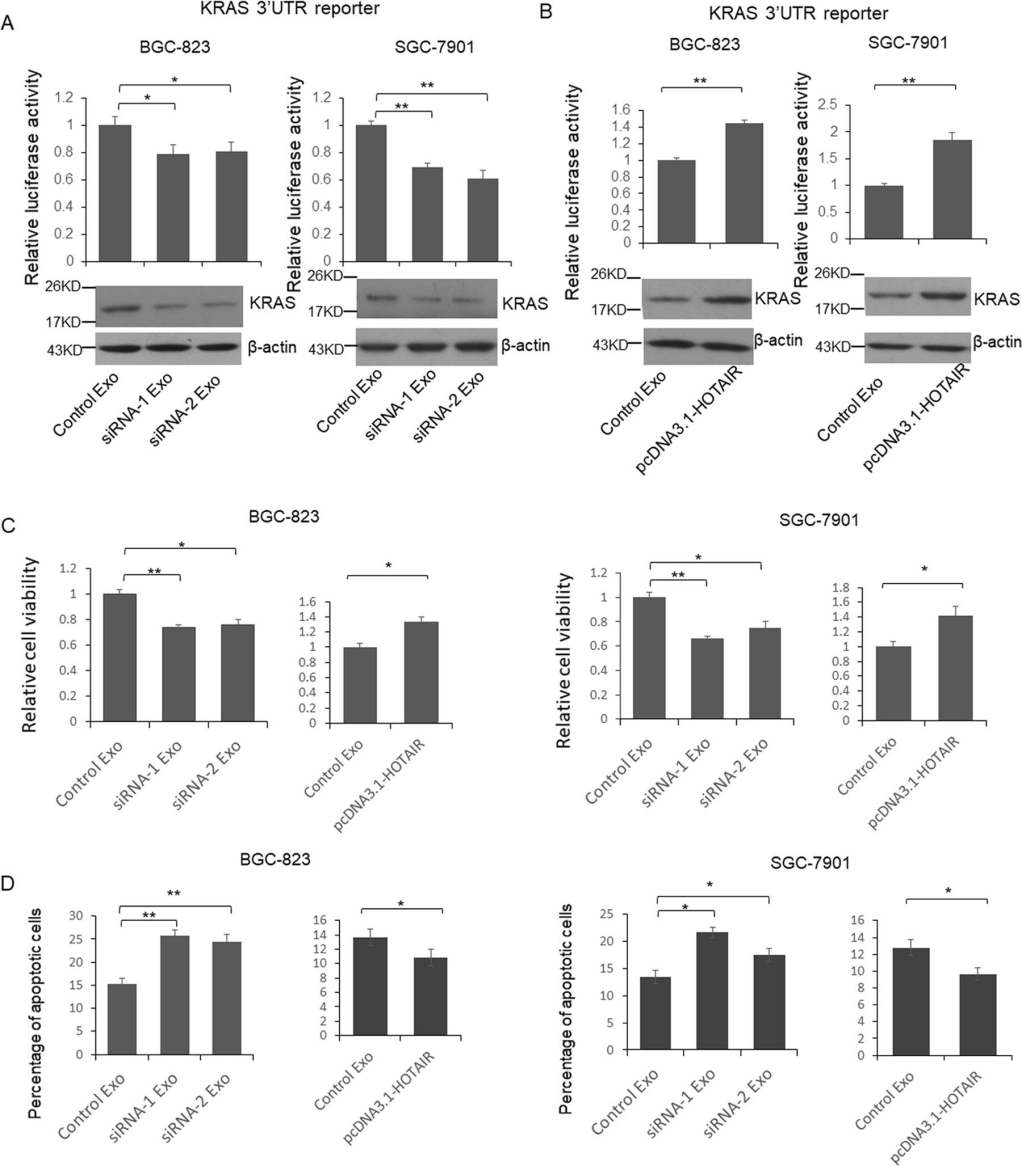
HOTAIR modulated proliferation and apoptosis through regulating exosomal miRNAs. **a, b** BGC-823 or SGC-7901 cells were transfected with reporter vector and then cultured with exosomes from HOTAIR knockdown or overexpressed BGC-823 cells. 48 h after co-culture, cells were lysed and luciferase activities were detected. **c**, **d** BGC-823 or SGC-7901 cells were cultured with exosomes from HOTAIR knockdown or overexpressed BGC-823 cells. 48 h after co-culture, cell proliferation and apoptosis were detected. Results between three groups were analyzed by One-way ANOVA, and results between two groups were analyzed by student’s *t*-test. *P* < 0.05 was considered statistically significant. *P < 0.05, **P < 0.01.

Exosomes were confirmed can deliver proteins and miRNAs to the recipient cells and modulate the microenvironment of tumor. To explore the function of exosomes secreted by the GC cells, GC cells were transfected by KRAS reporter vector and then co-cultured with exosomes isolated from GC cells transfected with HOTAIR siRNAs or overexpression vector. Forty-eight hour after transfection, cells were lysed and the cell lysates were subjected to luciferase assay. As shown in Fig. 5a, the relative luciferase activity was significantly repressed in the cells co-cultured with exosomes from HOTAIR knocked down BGC-823 cells. Meanwhile, when HOTAIR overexpressed, the luciferase activities were significantly upregulated, suggesting functional miRNAs delivered via exosomes. In the same time, the co-cultured GC cells’ viability and apoptotic cell number were detected. As shown in Fig. 5c, the viability of GC cells was significantly repressed by exosomes from HOTAIR knocked down cells and upregulated by exosomes from HOTAIR overexpressed cells. Meanwhile, the apoptotic cells number was increased by exosomes from HOTAIR knocked down cells and decreased when co-cultured with exosomes from HOTAIR overexpressed cells (Fig. 5c).

To confirm the exosomes’ function on modulating KRAS expression, cell viability, apoptosis, migration, and invasion through the interaction between miR-30a/b and HOTAIR, we constructed HOTAIR knockout BGC-823 and SGC-7901 cells. HOTAIR knockout BGC-823 and SGC-7901 cells were transfected with KRAS reporter vector and then co-cultured with control exosomes or exosomes from miR-30a and -b antagonists treated SGC-823 cells. The luciferase activity was increased suggesting upregulated KRAS expression (Fig. S3A). When HOTAIR knockout BGC-823 and SGC-7901 cells were co-cultured with exosomes from miR-30a and -b antagonists treated SGC-823 cells, the cell viability (Fig. S3B) and apoptosis (Fig. S3C) were not significantly changed but the cell migration(Fig. S3D) and invasion (Fig. S3E) were upregulated. These results indicated that exosomes’ function on modulating cell viability and apoptosis was totally depend on the interaction between miR-30a/b and HOTAIR, but their function on cell migration and invasion was only partially relates to the interaction between miR-30a/b and HOTAIR.

### HOTAIR protected vascular endothelial growth factor A (VEGF-A) expression from the repression of miR-30a/b

It is identified that VEGF-A is a direct target of miR-30a and -b. To understand the function of HOTAIR in endothelial cells which involved in the tumor microenvironment, VEGF-A reporter vector was transfected into HUVEC cells with HOTAIR expression vector or the mixture of miR-30a and -b. As shown in Fig. S4A, HOTAIR upregulated VEGF-A expression in HUVEC cells, while, miR-30a and -b partially repressed the HOTAIR function on VEGF-A. Subsequently, the biological function of HOTAIR on HUVEC cells proliferation (Fig. S4B) and (Fig. S4C) capillary formation were examined. The results indicate that HOTAIR promoted proliferation and capillary formation in HUVEC cells, while miR-30a and -b totally repressed the HOTAIR function on proliferation and partially on capillary formation.

### HOTAIR controlled exosomal miR-30a/b level and regulated capillary formation via modulating VEGF-A expression

**Fig. 6:**
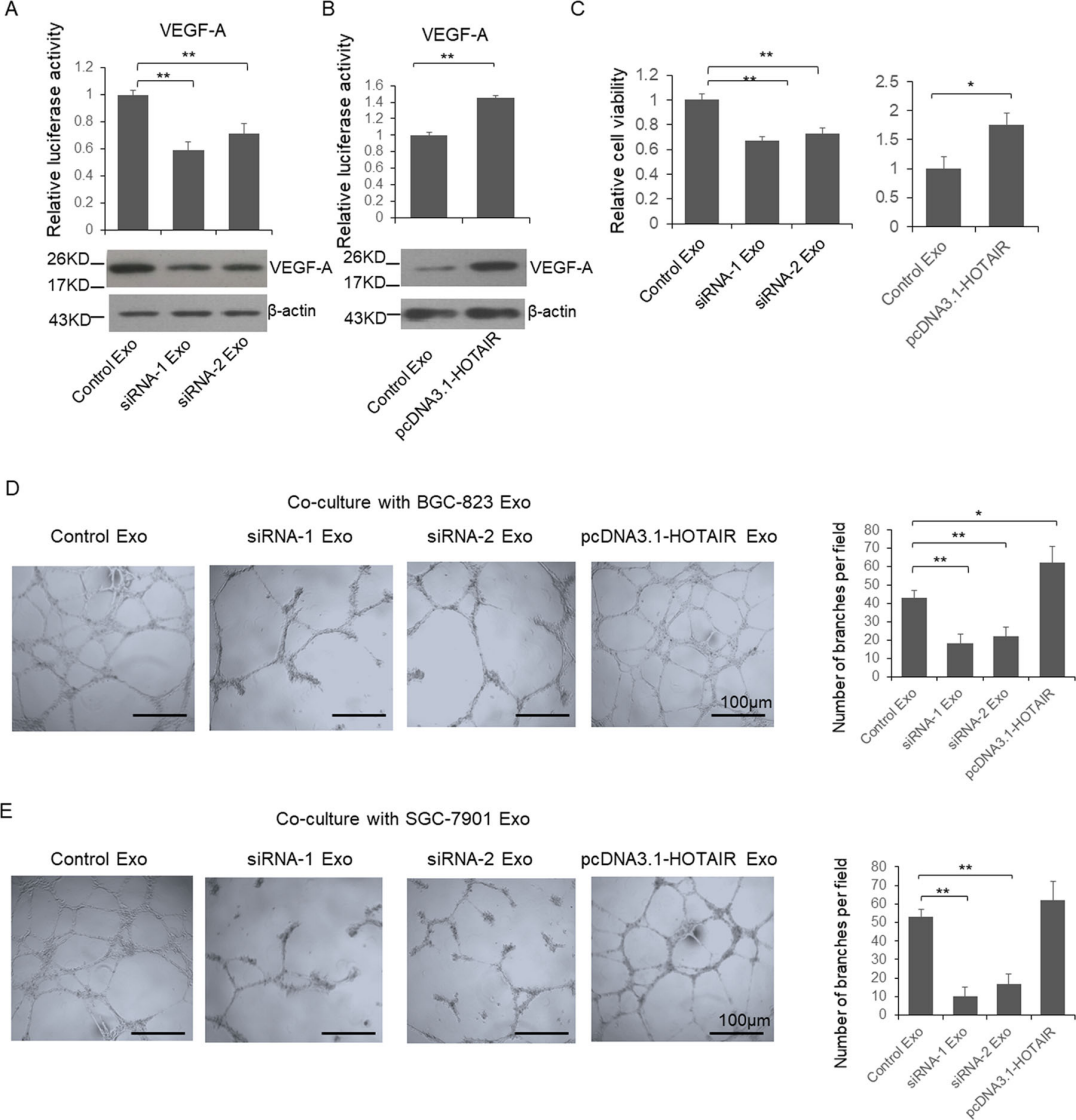
HOTAIR modulated the angiogenesis through altering exosomal miRNA. HUVEC cells were transfected with reporter vector and then cultured with exosomes from HOTAIR knockdown (**a**) or overexpressed (**b**) BGC-823 cells. Forty-eight hour after co-culture, cells were lysed and luciferase activities were detected. **c**–**e** HUVEC cells were cultured with exosomes from HOTAIR knockdown or overexpressed BGC-823 or SGC-7901 cells. Forty-eight after co-culture, cell viability was detected. Meanwhile, capillary formation assay was used to determine the angiogenetic capacity of HUVEC cells. Results between two groups were analyzed by student’s *t*-test, and results between more than two groups were analyzed by One-way ANOVA. *P* < 0.05 was considered statistically significant. *P < 0.05, **P < 0.01.

To further unveil the effects of exosomes delivered by GC cells on the tumor microenvironment, HUVEC cells were transfected by VEGF-A reporter vector and then co-cultured with exosomes. As shown in Fig. 6a, the luciferase activity was reduced significantly in the cells co-cultured with exosomes from HOTAIR knockdown cells; meanwhile, the luciferase activity was increased by the exosome from HOTAIR overexpression cells (Fig. 6b). Consistently, the cell viability was repressed by exosomes from HOTAIR knockdown cells and increased by HOTAIR overexpression cells (Fig. 6c). Subsequently, HUVEC cells were co-cultured with exosomes and capillary formation assay was used to determine the capacity of angiogenesis. As shown in Fig. 6d, e, the number of capillary was significantly reduced when HUVEC cells co-cultured with exosomes from HOTAIR knockdown GC cells. Meanwhile, when co-cultured with exosomes from HOTAIR overexpressed cells, the number of capillary was upregulated.

To confirm the exosomes’ function on VEGF-A expression, viability and capillary formation capacities of HUVEC cells depend on the interaction between miR-30a/b and HOTAIR, we constructed VEGF-A knockout HUVEC cell. HOTAIR knockout HUVEC cells were transfected with VEGF-A reporter vector, and then co-cultured with control exosomes or exosomes from miR-30a and -b antagonists treated SGC-823 cells. The luciferase activity (Fig. S5A), cell viability (Fig. S5B), and capillary formation (Fig. S5C) capacity were not significantly changed indicating the exosomes’ function on VEGF-A expression, viability and capillary formation capacities of HUVEC cells totally depend on the interaction between miR-30a/b and HOTAIR.

### Exosomes from HOTAIR knockdown GC cells inhibit tumor growth in vivo

**Fig. 7:**
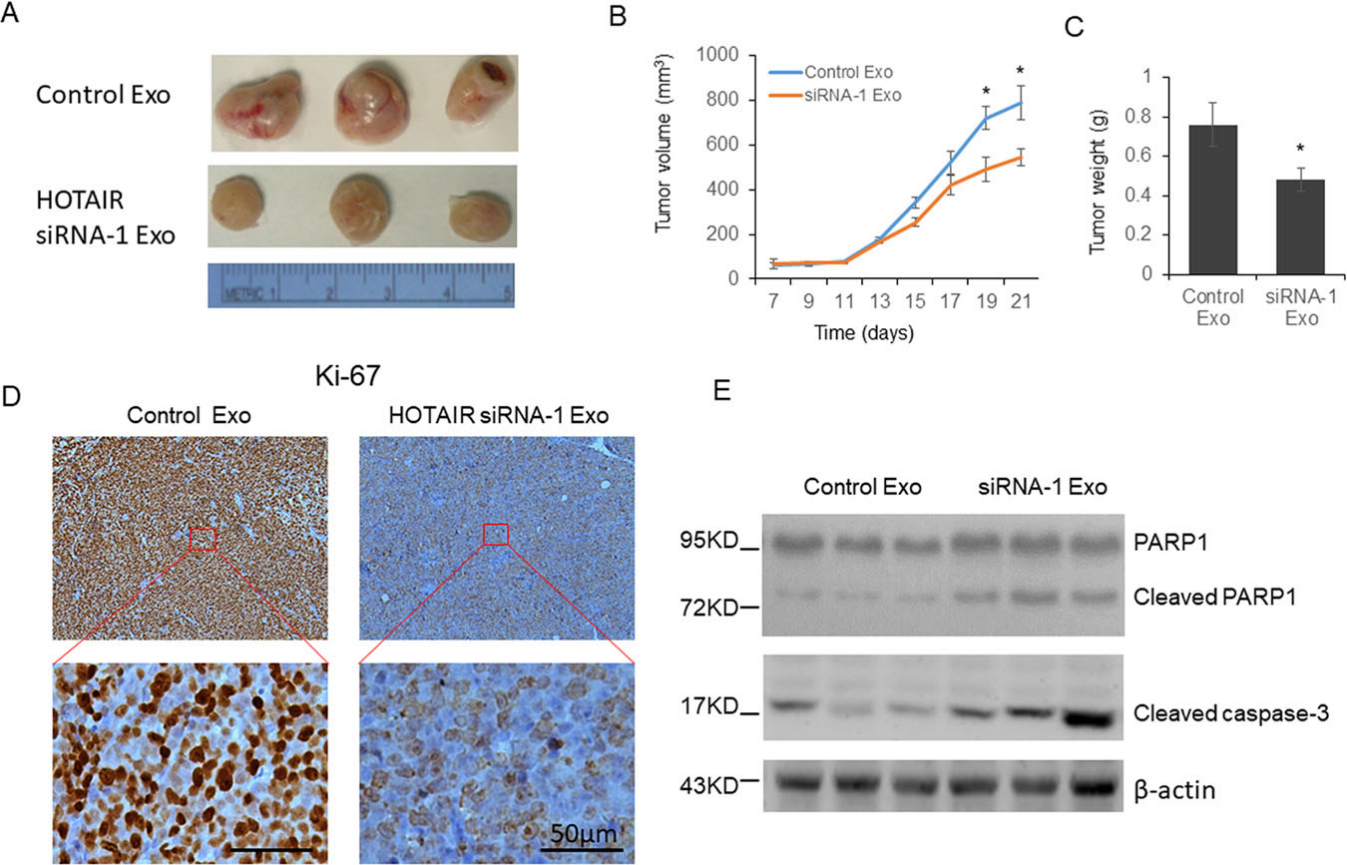
Exosomes from HOTAIR knockdown GC cells repressed GC tumorigenesis. A total of 5 × 10^6^ BGC-823 cells in were injected subcutaneously into the shoulder to establish a human GC cell xenograft mouse model. Exosomes were injected directly into the tumor at days 14, 21, 24, and 28 after tumor cell injection. The Ki-67 level was determined by immunohistochemistry, and the level of cleaved PARP1 and caspase-3 was detected by immunoblotting. Results were analyzed by student’s *t*-test. *P* < 0.05 was considered statistically significant. *P < 0.05, **P < 0.01.

To further examine the function of exosomes in vivo, BGC-823 cells were injected into shoulder of SCID mice to establish a human MM xenograft model. At 14 days after tumor cell injection exosomes from HOTAIR knockdown BGC-823 cells were injected directly into the tumor and again at days 21, 24, and 28. We observed reduced tumor size and weight in the siRNA-1 exosome injection group (Fig. 7a–c). At the same time, decreased Ki-67 was observed in the tumor section (Fig. 7d), and increased cleaved PARP1 and caspase-3 was found in siRNA-1 exosome injection group (Fig. 7e).

## Discussion

Due to the advancement of medical technology, the mortality of gastric cancer have gradually decreased, but there are still nearly 700,000 confirmed mortalities annually worldwide (4). Furthermore, a large number of patients are diagnosed with advanced gastric cancer and have a poor prognosis. Thus, more sensitive markers for GC early stage diagnosis, tumor grading, prognostic evaluation after treatment is urgently needed. It is reported that a large group of noncoding RNAs are dedicated to keeping the homeostasis of the cell and body^9–11^. Abnormal noncoding RNA expression has been confirmed in the clinical specimens of patients with many kinds of malignancies, including GC^30,31^. In this study, we first detected the expression of 11 selected miRNAs in the exosomes from 87 GC patients’ plasma samples and found four miRNAs (miR-30a, -30b, -222, and -16) level was significantly altered. Among these miRNAs, miR-222 level has been identified upregulated in the plasma samples of GC patients by two different groups^32,33^. Zhang J et al. reported that miR-16 was downregulated in the plasma samples from patients with GC and related to poor prognosis^34^. In this study, our results support the biomarker potential of circulating miR-16 and miR-222 for GC diagnosis.

MiR-30a and -b have been found downregulated in GC tissue samples and function as tumor suppressors^35–37^. In the present study, we report the downregulated exosomal miR-30a and -b in GC patients for the first time. However, in 2017, Wang N et al. examined the exosomal miR-30a level in serum samples from GC patients and did not found a significantly difference when compared with healthy controls^38^. The inconsistency may be caused by different cohorts or sample origin (serum or plasma), so the expression pattern of miR-30a and -b in circulation needs to be further confirmed.

Through analyzing the expression of four miRNAs and four lncRNAs, we found weak negative correlations between the GC tissue HOTAIR level and plasma exosomal miR-30a, -b and -16 level. Interestingly, strong negative correlations were found between the GC tissue HOTAIR level and exosomal miR-30a, -b, and -16. HOTAIR has been reported can function as a ceRNA binds with several miRNAs in GC tissues including miR-331^26^, miR-217^39^, miR-126^19^ et al. In the present study, we identified the direct interaction between HOTAIR miR-30a, -b, or -16 in GC cells for the first time. There are reports indicated that the extensive base pairing between miRNAs and target mRNAs can promote miRNA decay^20^. But it is still unknown whether the interaction between miRNA and HOTAIR induces instability of miRNAs. In this study, we provide the evidences that HOTAIR can induce the degradation of miR-16, miR-30a, and miR-30b in GC cell lines, which unveiled a new mechanism of how HOTAIR regulates miRNAs’ function.

The tumor microenvironment (TME) plays an important role during tumor growth and metastatic progression, and HOTAIR has been found function as a key regulator of the TME. For example, endothelial cells are responsible for supporting tumor neovasculature, also participating in several molecular signaling pathways, and HOTAIR has been identified can regulate VEGF-A and angiopoietin 2 (Ang2) expression in nasopharyngeal carcinoma and glioma^40,41^. VEGF-A is a signal protein produced by cells that stimulates angiogenesis confirmed. Meanwhile, VEGF-A is also a confirmed miR-16 target gene^42^. In the present study, we identified that GC cellular HOTAIR level can modulate the capillary formation of endothelial cells via regulating the exosomal miRNAs, indicating a new oncogenetic function of HOTAIR during the pathogenesis of GC.

KRAS has been identified as a direct target of miR-30 family members^43^. Since exosomes can also transmit materials between tumors cells, we detected the function of exosome delivered miR-30a and -b on GC cells proliferation and apoptosis. Determined by luciferase reporter system, the expression of KRAS in GC cells was reduced or increased when co-cultured with exosomes from HOTAIR knockdown or overexpressed GC cells. Meanwhile, when co-cultured with exosomes from HOTAIR knockdown GC cells, GC cells exhibited reduced cell viability and increased apoptosis. Because inherent variations exist not only between patients with same type of malignancies but also within any individual tumors^44^, so we believe that HOTAIR may play an important role on regulating intratumor communications between different type GC cells.

There are still some weaknesses of this study. The first one is, although we found four altered functional miRNAs in GC patients’ plasma, it is still difficult to identify where are these miRNAs from and where are they going to in vivo. Further in vitro tissue culture and in vivo methods need to be used to track their paths and decipher their function during the development of GC. The second one is, our functional study on miR-30a/b just depends on two confirmed miR-30a/b targets, VEGF-A and KRAS. However, a single miRNA usually has tens to hundreds direct targets, so the full scale function of miR-30a/b in tumor and TME is still unclear. Further research depend on RNA immunoprecipitation (RIP) and microarray or high-throughput sequencing will be helpful to examine the roles of miR-30a/b during the progression of GC.

In conclusion, we report the correlation between the plasma miRNAs level and GC tissue HOTAIR expression for the first time. We confirmed that HOTAIR regulated both cellular and exosomal miRNAs expression by direct binding and inducing degradation. These findings unveiled the oncogenic role of HOTAIR during the tumorigenesis of GC and may provide new biomarkers for GC clinical diagnosis and treatment.

## Supplementary information

Figure s1

Figure s2

Figure s3

Figure s4

Figure s5

Table s1

## Data Availability

The datasets used and/or analyzed during the current study are available from the corresponding author on reasonable request
