# Supplementary figures and images for: HOTAIR contributes to the carcinogenesis of gastric cancer via modulating cellular and exosomal miRNAs level
Source: Cell Death Dis. 2020 Sep 19;11(9):780. doi: 10.1038/s41419-020-02946-4 (PMC7502082; doi:10.1038/s41419-020-02946-4)

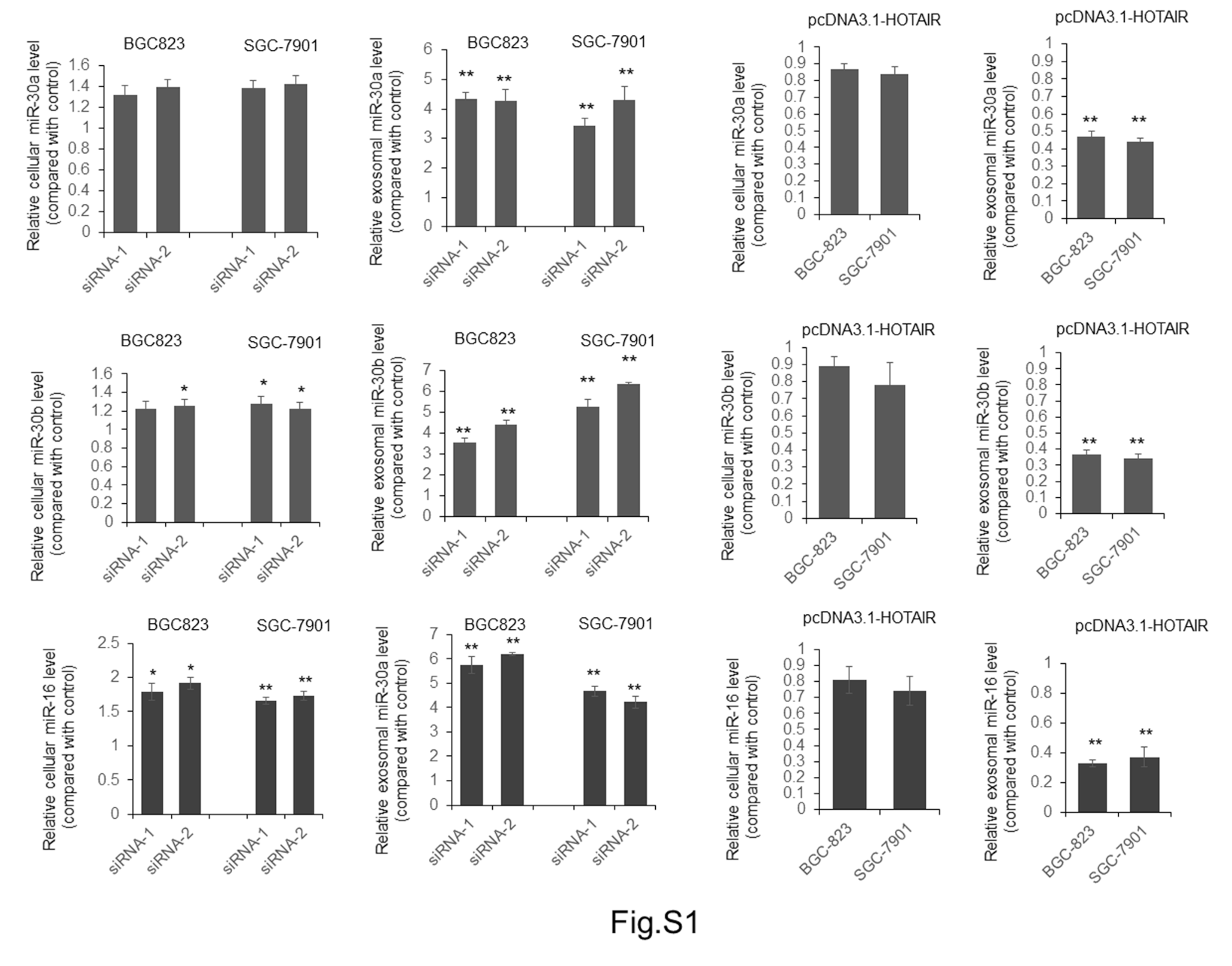

Supplement: Supplementary file 1 — Figure s1 [file 41419_2020_2946_MOESM1_ESM.tif]

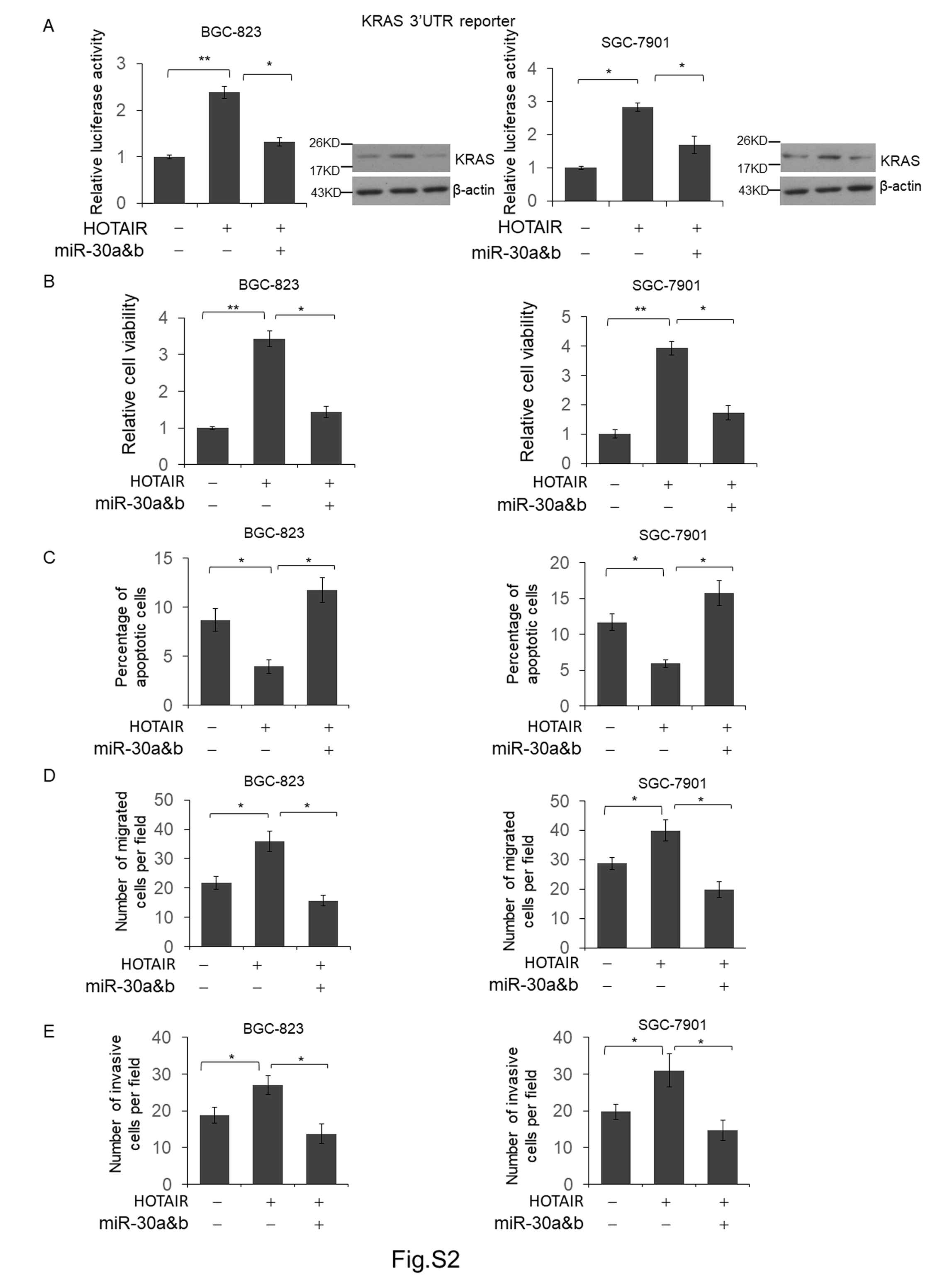

Supplement: Supplementary file 2 — Figure s2 [file 41419_2020_2946_MOESM2_ESM.tif]

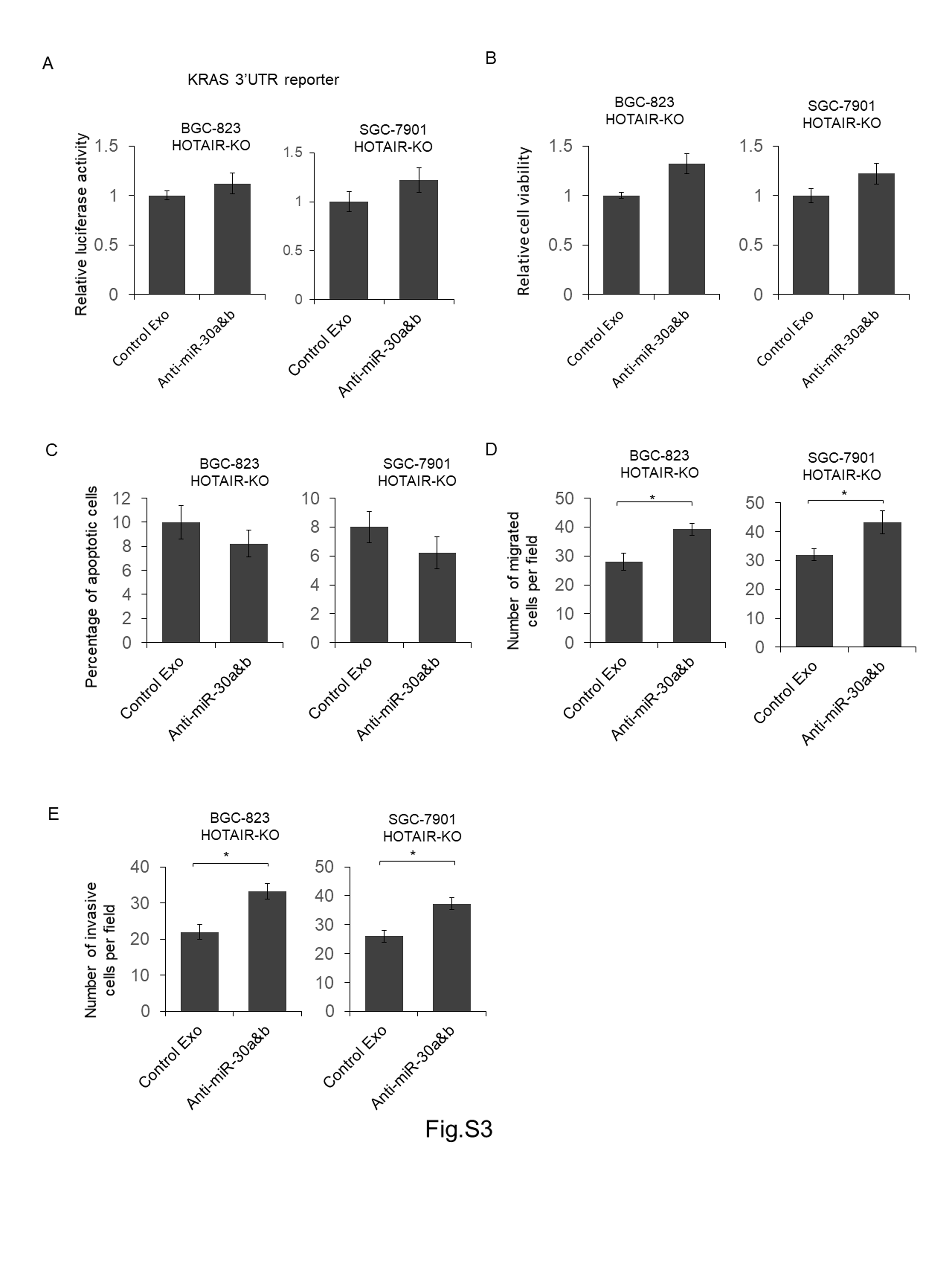

Supplement: Supplementary file 3 — Figure s3 [file 41419_2020_2946_MOESM3_ESM.tif]

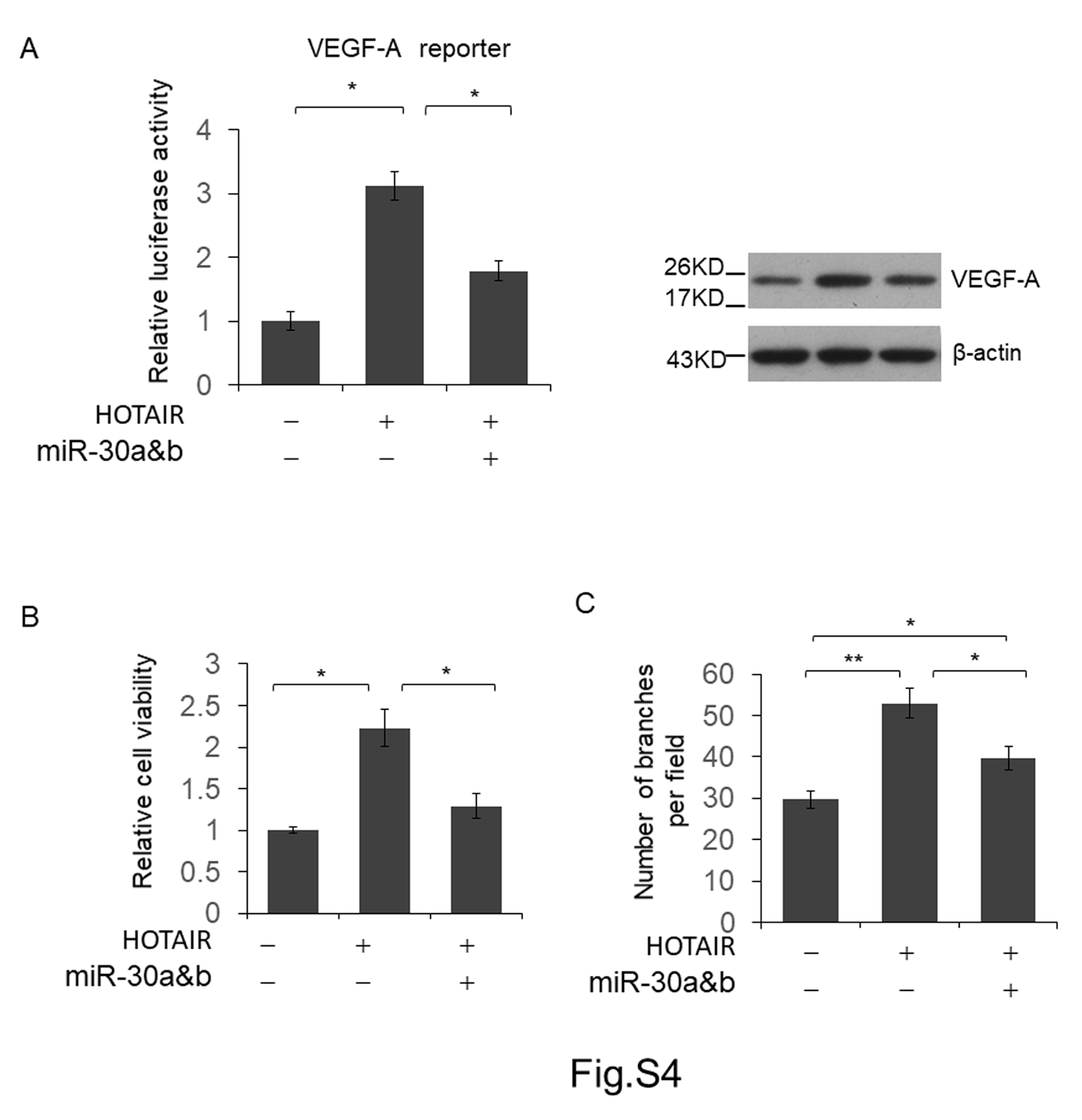

Supplement: Supplementary file 4 — Figure s4 [file 41419_2020_2946_MOESM4_ESM.tif]

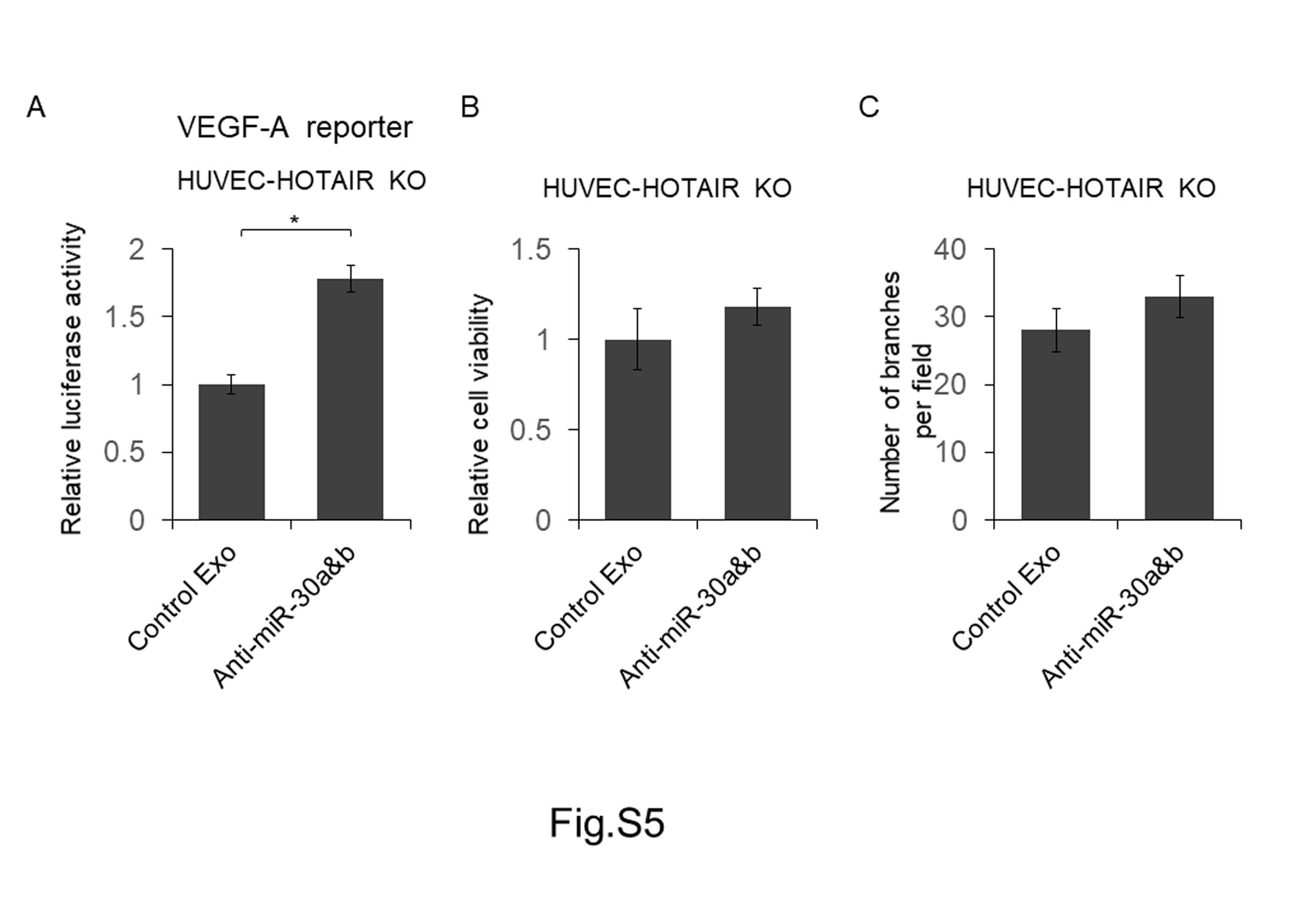

Supplement: Supplementary file 5 — Figure s5 [file 41419_2020_2946_MOESM5_ESM.tif]
